# The relationship between Zoster serology, vaccination uptake and infection rates: a single-centre cross-sectional study

**DOI:** 10.1093/rap/rkae127

**Published:** 2024-10-08

**Authors:** Saad Ahmed, Martin Lauran, Adaeze Ugwoke, Tom Walton, Chris Holroyd, James Galloway

**Affiliations:** Department of Rheumatology, Addenbrookes Hospital, Cambridge, UK; Centre for Rheumatic Disease, King’s College London, London, UK; Department of Rheumatology, Addenbrookes Hospital, Cambridge, UK; Department of Rheumatology, Colchester Hospital, Essex, UK; Rheumatology Department, University Hospital Southampton NHS Foundation Trust, Southampton, UK; Centre for Rheumatic Disease, King’s College London, London, UK

**Keywords:** Herpes Zoster, vaccination, Janus kinase inhibitors

## Abstract

**Objective:**

This study aimed to assess the prevalence of Varicella-Zoster Virus (VZV) immunity, vaccination uptake and incidence of VZV-related events in inflammatory arthritis (IA) patients initiating biologic or targeted synthetic disease-modifying antirheumatic drugs.

**Methods:**

An observational study was conducted in a single hospital between March 2019 and December 2020. Ninety-three IA patients were included. Data were collected from electronic health records and analysed using the chi-squared test.

**Results:**

The majority of patients (91.4%) were seropositive for VZV, reaffirming the necessity for vaccination. In total, 8.6% of the cohort received the Zostavax vaccine, despite a small yet significant number of patients (4.3%) experiencing Herpes Zoster after initiating treatment. Multiple factors contributed to low vaccine uptake, including limited vaccine availability, discrepancies between the British Society for Rheumatology and Joint Committee on Vaccination and Immunisation guidelines, vaccine hesitancy and concerns regarding vaccine efficacy and risks.

**Discussion:**

Significant VZV immunity exists among patients prior to targeted therapy commencement. Risk factors for VZV-related events include Janus kinase inhibition, increasing age and long-term steroid use. VZV-related events occurred exclusively in patients with prior viral immunity. Despite most patients having serological evidence of prior VZV exposure, our study exposes critical gaps between current clinical guidelines and practice, particularly in VZV vaccine uptake. Barriers to vaccination include inconsistent guidelines, limited vaccine availability and patient-level hesitancy. This is concerning as our cohort demonstrated small but significant rates of zoster, mostly among patients on long-term steroids.

Key messages

- A greater risk of Herpes Zoster infection has been observed in patients with an inflammatory arthritis treated with JAK inhibitors, a risk further increased with long-term steroid treatment.
- Vaccination against Herpes Zoster targets viral reactivation rather than initial infection.
- A low uptake rate of Herpes Zoster vaccination was observed, largely due to prior guideline discrepancy, in our cohort.

## Introduction

Herpes Zoster (HZ), colloquially known as ‘shingles’, is a condition resulting from the reactivation of the Varicella-Zoster Virus (VZV) [1]. Initial exposure to VZV typically leads to chickenpox, and the virus subsequently establishes latency within the dorsal root ganglia of spinal or cranial nerves. The re-emergence of this dormant virus manifests as shingles, which is marked by a painful, localized rash [2]. Complications can range from persistent post-herpetic neuralgia (PHN) to vision-threatening Herpes Zoster Ophthalmicus [3].

In the UK, over 80% of VZV primary infections occur during childhood, making shingles more prevalent in middle-aged and older adults. The incidence notably increases for individuals over the age of 50, likely due to immune system ageing [4, 5]. Various risk factors contribute to the occurrence of HZ, such as gender, ethnicity and comorbidities such as autoimmune diseases and diabetes mellitus [6, 7]. Specific medications, including Janus kinase (JAK) inhibitors, a recent class of orally administered molecules targeting multiple immune pathways, have been shown to significantly elevate HZ risk compared with other immunosuppressants [8–11].

Two vaccines for shingles, Zostavax and Shingrix, have been licensed in the UK [12]. While Zostavax reduces the incidence of shingles by half in those over 60 [13], Shingrix offers superior effectiveness and has been recently introduced to the UK market [14–16]. Nonetheless, limited availability has led to strict eligibility criteria [17]. Guidelines from the Joint Committee on Vaccination and Immunisation (JCVI) and the British Society for Rheumatology (BSR) offer contrasting recommendations on vaccination eligibility, particularly for patients initiating biologic DMARDs [18].

Furthermore, Zostavax, being a live attenuated vaccine, means its use is contraindicated in primary and acquired immunodeficiency states due to the risk of causing VZV-related diseases, thus limiting use in our patient population. Shingrix is a recombinant zoster vaccine and therefore does not contain the live virus, making it a safer alternative for immunosuppressed patients, though its higher cost means a more gradual national rollout programme is occurring in the UK.

The current BSR guidelines recommend vaccination with Zostavax in all patients above the age of 50 prior to biologic commencement, unless a Shingles episode occurred in the last year; an update of the guideline is currently underway. During the study time period, the JCVI recommended vaccination against HZ in persons aged above 70 years old only.

This research aims to (1) quantify the prevalence of VZV immunity among patients before the initiation of biologic or targeted therapies, (2) assess the adoption rate of VZV vaccination, and (3) examine the frequency of new VZV events post-treatment initiation. Possible predictors affecting VZV immunity may also be evaluated. Data were collected through a cross-sectional analysis of routine care as part of a local service evaluation.

## Methods

### Study setting and participants

This research employed a cross-sectional observational design and was executed in a single district general hospital in England from March 2019 to December 2020. We focused on adult patients (age > 18 years) diagnosed with an inflammatory arthritis (IA) who were newly prescribed either a biologic or targeted synthetic disease-modifying anti-rheumatic drug (bDMARD or tsDMARD). Participants were selected through electronic health record (EHR) data gathered from a regional database tracking patient visits to rheumatology clinics. There were no exclusion criteria in our study.

### Data sources and validation

A portion of the participant pool had vaccination histories available, extracted from primary care EHRs. These histories were cross-verified using hospital episode statistics and electronic summaries of hospital admissions.

### Variables and data collection

We assessed predictor variables that included patient age, specific diagnosis and long-term use of steroids (characterized as >5 mg oral prednisolone for over a month). The outcome variables were VZV serology, incidence rates of HZ and PHN, and uptake of HZ vaccines. Laboratory tests, part of routine care, were used to determine prior exposure to VZV, although not every patient underwent testing. Data on chickenpox exposure, HZ episodes and PHN were collected from the EHR and direct clinical consultations. Additional demographic and prescribing data were sourced from the Trust’s EHR and the pharmacy’s electronic database, respectively.

### Statistical approach

The data, extracted on 28 July 2021, underwent analysis using Stata MP 17.0. Continuous variables are presented as means with standard deviations, and categorical variables are depicted in numbers and percentages. Given the study’s cross-sectional nature, the primary analysis was descriptive. We employed the Chi-squared test to explore the relationships between patient characteristics and the completeness of zoster screening prior to therapy initiation. To mitigate sampling bias, the study incorporated all eligible patients who attended a rheumatology clinic within the study timeframe. Although missing data were acknowledged, the small sample size precluded any statistical modelling for these missing data points.

### Ethical considerations

The study received internal approval and was conducted as part of a local service evaluation.

## Results

### Patient demographics and treatment characteristics

A total of 93 patients participated in this single-centre, cross-sectional study spanning March 2019 to December 2020. The largest group of patients had Rheumatoid Arthritis (RA, *n* = 50), followed by Axial Spondyloarthritis (AxSpA, *n* = 25) and Psoriatic Arthritis (PsA, *n* = 18). The mean age at the initiation of bDMARD treatment was 57 years for the RA group, 44 years for the AxSpA group and 48 years for the PsA group. For the overall cohort, the mean age was 52 years. Anti-TNF inhibitors were the most frequently prescribed medication, constituting 62% of the cohort, with JAK inhibitors used by 10%. Long-term steroid usage, defined as over 5 mg of oral prednisolone for more than a month, was observed in 28% (*n* = 26) of the patients. Sixteen of the 93 patients studied were on concomitant Methotrexate at an average dose of 20 mg once weekly. Only four of these patients had VZV serology assayed, and all were positive; the serostatus of the other 12 patients is unknown. None of the 16 patients had Shingles or PHN during treatment with Methotrexate or targeted therapies. Table 1 shows a summary of the demographic and results data.

**Table 1.** Demographic characteristics and results summary

Number of participants		Average age of starting DMARD (*P* < 0.001)	Number of participants per rheumatological disease (*P* < 0.001)			Number of participants per medication class (*P* < 0.001)							VZV positive participants (*P* = 0.8)	VZV Negative participants	Patients on long term steroid treatment (*P* < 0.001)	Participants with history of VZV-related events prior to bDMARD (*P* = 0.93)	Participants who received Zostavax (*P* = 0.12)	Participants with Shingles while on bDMRD (*P* = 0.17)	Participants who developed PHN (*P* = 0.42)
Over 40	Under 50	52	RA	PsA	AxSpA	TNFi	CTLA4 blockers	Anti CD20	Jaki	Anti IL6	IL 12-23	IL17	85	6	26	87	8	4	2
37	56		57	48	44	58	3	8	9	4	3	7							

DMARD: Disease-modifying anti rheumatic drug; RA: Rheumatoid Arthritis; PsA: Psoriatic Arthritis; AxSpA: Axial Spondyloarthritis; TNFi: TNF inhibitors; JAKi: Janus kinase inhibitors; VZV: Varicella-Zoster Virus; bDMARD: biologic DMARD; PHN: post-herpetic neuralgia.

### VZV screening and vaccination

Current BSR guidelines [18] advocate VZV antibody testing for patients with uncertain or negative VZV history prior to biologic therapy. Nonetheless, none of the six patients who self-reported being VZV-naive underwent serology tests or received vaccinations. Additionally, only 8 out of 55 eligible patients above the age of 50 were vaccinated against HZ as per BSR guidelines.

### VZV serology and patient recall

Patient recall for a history of chickenpox or shingles aligned closely with VZV serology results; 93.5% (*n* = 87) reported prior exposure, and 91.4% (*n* = 85) tested positive for VZV antibodies. Two patients from the self-reported positive cohort did not undergo VZV serology testing. Ninety-one patients were tested for VZV serology.

### VZV incidence and post-treatment outcomes

Four patients (4.3% of the cohort) developed shingles after starting bDMARD treatment; all were VZV-positive and diagnosed with RA. Three of these were on long-term steroids. Two patients (50% of the shingles cases) subsequently suffered from PHN; both were VZV-positive, and one was on long-term steroids. Table 2 shows relevant demographic and clinical data for the Shingles patient cohort.

**Table 2.** Characteristics of Shingles cohort

Diagnosis	bDMARD	Vaccinated with Zostavax (or other prophylaxis received)	Long term steroids	Age of starting Biologic	Duration of bDMARD therapy (months)	Shingles episode	PHN	Time interval between bDMARD commencement and Shingles episode (months)
RA	Rituximab	No	Yes	85	18	Yes (and HZO)	No	1
RA	Tofacitinib	No	Yes	63	15	Yes	No	6
RA	Adalimumab	No	No	57	18	Yes	No	3
RA	Etanercept	No	Yes	55	18	Yes	Yes	8

bDMARD: biologic DMARD; PHN: post-herpetic neuralgia; HZO: Herpes Zoster Ophthalmicus; RA: Rheumatoid Arthritis.

### Vaccine uptake

The overall vaccine uptake was low. Only 8.6% received Zostavax prior to initiating advanced therapy, while none had received Shingrix. Adherence to JCVI guidelines, which recommend vaccination only for those over 70 years, might partially explain this low uptake. In our cohort, adherence to vaccination guidelines from JCVI and BSR was 42.1 and 14.5%, respectively, indicating a significant shortfall in meeting the recommendations of both guidelines. In our cohort, the percentage of patients eligible for vaccination against HZ according to the JCVI guidelines at the time was 19.35 and 39.78% according to BSR. The findings reveal poor adherence to established guidelines for VZV screening and vaccination, particularly in patients over 50 and those initiating advanced therapies.

## Discussion

### Key findings

Our study offers crucial insights into VZV immunity and related complications in patients with an IA undergoing treatment with bDMARDs or tsDMARDs. Three key observations stand out: first, most patients had prior VZV exposure, underscoring the necessity to vaccinate against reactivation, rather than initial infection. Second, we observed a troublingly low rate of vaccine uptake. Lastly, although the sample showed small numbers, there was a significant incidence of HZ activation, emphasizing the real-world implications of this clinical issue.

Our centre’s policy is to advocate testing for VZV antibody in patients without a personal history of chickenpox rather than test all patients prior to bDMARD commencement. This is in line with the BSR guidance. At the time of this study, we did not have a biologics proforma, but now do and everyone gets tested. We do not feel that national guidance should advise VZV serology assays in all patients. Chickenpox and Shingles are due to the same virus. It is unknown whether Zostavax protects against chickenpox in patients with no prior history but it likely does as it uses the same virus strain as the childhood chickenpox vaccine, Varivax.

Since September 2021, Shingrix has been available to immunosuppressed patients aged 70 and above in whom Zostavax is contraindicated. From September 2023, the Shingrix vaccine eligibility has expanded to include immunosuppressed persons aged above 50. The Shingrix vaccine will gradually replace Zostavax for the UK shingles vaccination programme, and immunosuppressed persons aged above 50 represent a high priority group, with no upper age limit to vaccination. The age of vaccination for immunocompetent persons will be lowered from 70 to 60 in a phased implementation programme over a 10-year period.

Potential reasons for lack of VZV antibody testing in patients with uncertain prior exposure may be due to lack of clinician familiarity with the 2019 BSR guidelines, lack of a local consensus biologic DMARD proforma and checklist, limited local primary care VZV testing capability, the recent implementation of electronic blood test requests and possibly the lack of a local DMARD initiation nurse led clinic.

### Factors hindering vaccine uptake

The low HZ vaccination rate in our study is in keeping with prior data, which reports rates to be as low as 10% in RA and PsA patients [19, 20]. The vast majority of our patients were seropositive for VZV (93.4%) and had developed chickenpox in the past, but this was not shown to be protective against subsequent Shingles or PHN. Serologic testing prior to HZ vaccination is in general not recommended given the high positive predictive value of a self-reported history of VZV and widespread exposure in older adults [21], our results support this. Previous studies have shown that strong predictors of HZ events include Asian ethnicity and prior HZ [22], but we could not corroborate these with our data.

The low vaccination rate can be attributed to multiple challenges, including limited vaccine availability, diverging guidelines from BSR and JCVI that create confusion, and general patient hesitancy towards vaccines. Additionally, clinicians’ express reservations due to the live vaccine’s limited efficacy and potential risks. At the time of our study, however, the key factor resulting in low vaccine uptake remains discordant national guidelines surrounding HZ vaccination in immunosuppressed patients.

Shingrix has a greater efficacy than Zostavax; rollout of Shingrix is still, however, underway and therefore clinician reservations concerning Zostavax and its potential risks currently remain. There is a risk of VZV-related disease with live vaccination, which can rarely be fatal, and this may further contribute to incomplete coverage.

Factors contributing to vaccine hesitancy are poorly understood but have decreased over the last few years [23] in rheumatic diseases. This is a particular issue with HZ with recent data showing only 50% of individuals demonstrating a willingness to be vaccinated [24]. Possible reasons include concern regarding vaccine efficacy, safety, cost and perceived risk of developing HZ. Psychosocial factors, including a sense of collective responsibility, also contribute to vaccine hesitancy [25]. The fifth and equally important factor is the widespread lack of awareness about the need for vaccination against VZV.

### Risk factors and clinical implications

The study reaffirms existing literature pointing to older age and long-term steroid use as significant risk factors for developing shingles, in addition to ethnicity, comorbidities and polypharmacy [26]. All instances of shingles occurred in patients who were VZV-seropositive and diagnosed with RA, of whom half developed PHN. This serves as a stark reminder of the critical importance of identifying those at elevated risk of VZV complications and adopting preventive measures.

### Limitations and future directions

Our study is limited by a small sample size, which impacts the reliability of our findings. Furthermore, the study did not explore potential variations in VZV risk across different medication classes or treatment regimens. These are areas suitable for future research. We plan to longitudinally follow up our patient cohort, which will provide real-world data on the long-term efficacy of vaccination in preventing HZ-related adverse events.

## Conclusion

In summary, our study highlights the urgent need to address the gap between clinical guidelines and real-world practice regarding VZV vaccination in patients with an IA. The low vaccine uptake, despite most patients having prior VZV exposure and a significant minority developing HZ, emphasizes the immediate need for unified guidelines, better vaccine availability and heightened awareness among both clinicians and patients. Addressing these issues could substantially mitigate the risks associated with VZV reactivation in this vulnerable population.

## Supplementary Material

rkae127_Supplementary_Data

## Data Availability

The data underlying this article are available in the article and its online supplementary material.
