# Supplementary material for: The relationship between Zoster serology, vaccination uptake and infection rates: a single-centre cross-sectional study
Source: Rheumatol Adv Pract. 2024 Oct 8;8(4):rkae127. doi: 10.1093/rap/rkae127 (PMC11513334; doi:10.1093/rap/rkae127)
Supplement: rkae127_Supplementary_Data [file rkae127_supplementary_data.docx]

**Supplementary Table S1: Characteristics of VZV negative cohort**

| Diagnosis | bDMARD | Vaccinated with Zostavax (or other prophylaxis received) | Long term steroids | Age of starting bDMARD | Duration of bDMARD therapy (months) | Shingles episode | PHN |
| --- | --- | --- | --- | --- | --- | --- | --- |
| RA | Rituximab | No | Yes | 53 | 14 | No | No |
| AxSpa | Adalimumab | Vzig | No | 58 | 11 | No | No |
| RA | Etanercept | No | Yes | 57 | 17 | No | No |
| RA | Rituximab | No | No | 52 | 9 | No | No |
| PsA | Secukinumab | No | No | 23 | 7 | No | No |
| AxSpa | Etanercept | No | No | 20 | 16 | No | No |


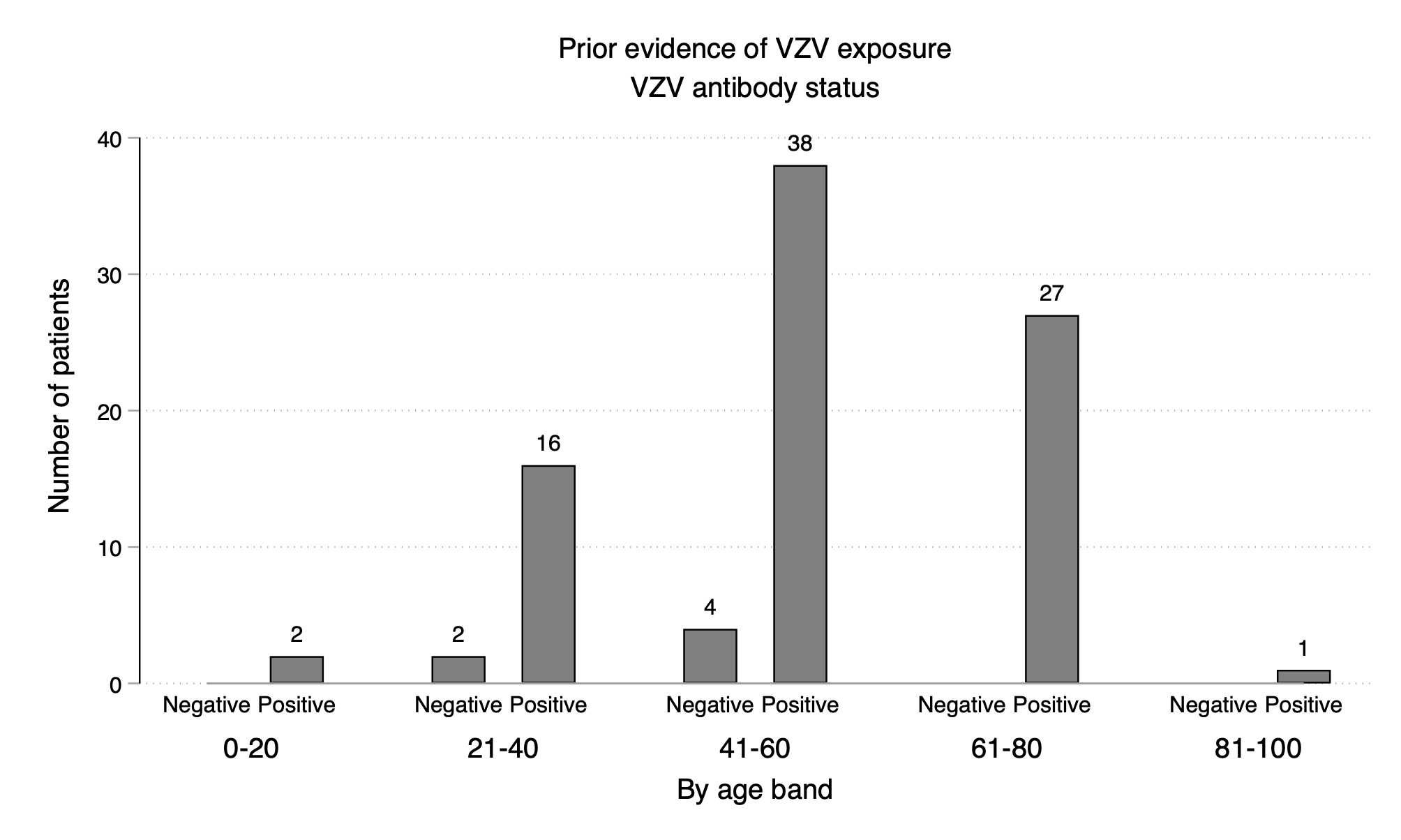


**Supplementary Figure S1: VZV antibody status according to age bracket.**
